# Systemic therapy of atopic dermatitis

**DOI:** 10.5414/ALX01285E

**Published:** 2017-08-04

**Authors:** C. Bußmann, N. Novak

**Affiliations:** Klinik und Poliklinik für Dermatologie und Allergologie, Universität Bonn, Germany; * N. Novak is being supported by a DFG NO454/5-2 Heisenberg professorship.

**Keywords:** atopic dermatitis, systemic therapy, antibiotics, immunotherapy

## Abstract

Therapy of severe atopic dermatitis, which is refractory to consistent treatment with topical steroids and topical calcineurin inhibitors is still a problem in many cases. The use of cyclosporine, which is the only approved systemic drug for the therapy of severe atopic dermatitis, is often limited by contraindications or adverse reactions. In this context, results from controlled and open-label studies with novel therapeutic approaches such as methotrexate, omalizumab or rituximab, which are in part very promising, are of great interest. In this work we would like to provide an overview of established and new therapeutic options for the treatment of severe atopic dermatitis.

German version published in Allergologie, Vol. 33, No. 4/2010, pp. 164-171

**Abbreviations:** AD: atopic dermatitis; CyA: cyclosporin A; FceRI: high-affinity IgE receptor; HND: head and neck dermatitis; IL: interleukin; LC: Langerhans cells; MTX: methotrexate; MMF: mycophenolate mofetil; M. sympodialis: Malassezia sympodialis; SCORAD: scoring atopic dermatitis; S. aureus: Staphylococcus aureus; SIT: allergen-specific immunotherapy; T reg: regulatory T cells; TPMT: thiopurine methyltransferase.

## Introduction 

Severe therapy-resistant atopic dermatitis (AD) defined by a SCORAD (Scoring Atopic Dermatitis) score of more than 35 – 40 is often associated with severe impairments in the patient’s private and professional life and leads to a significant impairment of their quality of life. 

In some cases, particularly in severe forms in adult patients, adequate stabilization cannot be achieved by avoidance of typical triggers, consistent basic therapy with lipid-regulating emollients, antiseptic or antibiotic measures or by regular therapy with topic steroids or topic immunomodulators. In these cases a systemic therapy is often considered. Before an immunosuppressant is applied, the hemogram as well as the liver, kidney and blood lipid values should be analyzed, and in unclear cases a biopsy should be performed in order to exclude T-cell lymphoma which could worsen under this therapy. Of course, severe internal diseases and malignomas should be excluded as well. 

Unfortunately, there are only few adequate drugs. Systemic steroids cannot be applied for longer periods due to their high risk of side effects, and the application of cyclosporine, the only immunosuppressant authorized for the systemic treatment of atopic dermatitis, is often limited by contraindications and side effects too. 

Recently some promising case series on alternative therapy approaches for AD using methotrexate, omalizumab or rituximab have been published. 

Thus, we would like to provide an overview of established and new therapeutic options for the treatment of severe atopic dermatitis. 

### Systemic corticosteroids 

Systemic steroids play an important role in the dermatological treatment as they have excellent anti-inflammatory and immunosuppressive effects. The binding of cortisol to intracellular glucocorticoid receptors leads to an increased transcription of the respective genes and thus to a suppressed expression of certain mediators like arachidonic acid and interleukin (IL)-1, IL-2, IL-3, IL-4 and IL-5. 

In AD the application of systemic steroids should be limited to exceptional cases. A 2- to 4-day pulse therapy or alternatively an application over 2 – 4 weeks with tapering off could be carried out [1]. 

A long-term therapy with internal steroids is contraindicated due to its side effects; thus, in many cases alternative forms of therapy are required [1]. 

## Antimicrobial therapy 

### Systemic antibiotics 

The skin of AD patients is densely colonized with Staphylococcus aureus (S. aureus). This germ frequently leads to superinfections of the eczematous skin lesions. S. aureus enterotoxins are both, allergens and superantigens, and have a proinflammatory effect which leads to the deterioration of the skin symptoms. Thus, antiseptic measures are an inherent part of treatment. 

Except in cases with severe superinfections the administration of systemic antibiotics has not been shown to result in clinical improvement and to have steroid-saving effects [17]. Therefore, systemic antibiotics should only be used in acute episodes with clear evidence for impetiginization in order to avoid resistances due to excessive application [15]. 

### Antimycotic therapy 

It has been suggested that the lipophilic yeast Malassezia sympodialis, which belongs to the normal skin flora and can be found mainly in the head and neck area, is a triggering factor for head and neck dermatitis (HND) [30]. In HND patients IgE-mediated sensitizations against this yeasts could frequently be demonstrated [45]. Furthermore it could be shown that the serum concentration of specific IgE against this lipophilic yeast correlates with the AD stage [23, 44]. 

Against this background, azoles, that have antimycotic and anti-inflammatory effects, were used in AD patients. In placebo-controlled studies a significant SCORAD reduction under itraconazole [51] and ketoconazole treatment [36] could be demonstrated. The antimycotic therapy approach that reduces the colonization with M. sympodialis, a potential trigger factor for HND, could therefore be a treatment option for this sub-form of AD. 

## Immunosuppressive therapy 

### Cyclosporin A 

Cyclosporin A is a cyclic peptide derived form the fungus Tolypocladium gams. Its immunosuppressive and anti-inflammatory effects are based on the inhibition of the enzyme calcineurin phosphatase that leads to an impaired release of IL-2 and to a suppression of T-cell proliferation. 

Reduced serum concentrations of the chemotactic mediator thymus- and activation-regulated chemokine (TARC/CCL17), of the soluble E-selectin and of the soluble CD30 are markers for the reduced recruiting of pro-inflammatory cells into the skin under CyA therapy [7, 14]. 

In addition, CyA has inhibitory effects on Langerhans cells [52]. Furthermore, the reduced T cell activity under CyA has an indirect reducing effect on keratinocyte apoptosis which is suggested to play a role in the pathophysiology, mainly in eczema development. 

It is assumed that CyA is particularly effective in AD patients in whom IgE autoreactivity plays a role as a trigger factor for eczemas. This effect could be due to the reduced T cell reactivity under CyA or based on a direct effect of CyA on autoreactive B cells [31]. 

Data concerning the effects of CyA on regulatory T cells (Treg) are controversial. One group of authors [8] could demonstrate an increase of Treg in the peripheral blood under therapy with low-dose CyA (2 mg/kg body weight (BW)), while other researchers [25] report a reduction of Treg in the blood of AD patients during therapy with 5 mg CyA per kg body weight. 

In Germany CyA is market-authorized for an interval therapy over a period of 6 months in patients over 18 years with severe therapy-resistant AD. Furthermore, a good tolerability could also be shown in children [5]. 

Numerous studies concerning the safety and efficacy of CyA in AD have been carried out. They could show that after 6 – 8 weeks the therapy starts to be effective and that the skin symptoms improve by at least 50% [5, 6, 11, 50]. 

Unfortunately, there are only few studies on the long-term therapy of AD with this immunosuppressant. The most important side effects of CyA are nephrotoxicity, muscle cramps due to magnesium deficiency, increased susceptibility to infections, increase in liver enzymes and in bilirubin, increase in blood lipid values, hypertension, gingival hyperplasia, disturbances of the central nervous system and changes in the hemogram. These side effects occur more frequently in adults than in children and depend on the dose [46]. 

There are two commonly used ways to start dosing. The manufacturers recommend a starting dose of 2.5 mg/kg BW which can be up-titrated to up to 5 mg/kg BW according to the patient’s response. A more rapid onset of efficacy is described for a starting dose of 5 mg/kg BW and a subsequent slow reduction until the lowest effective dose is reached [46]. 

One problem of CyA therapy is the frequently described recurrence of symptoms after discontinuation of therapy; in 8% of cases even a rebound effect can occur [46]. 

In Germany CyA is the only immunosuppressive agent with market authorization as a second-line treatment of severe therapy-resistant AD; all other systemic therapies can only be prescribed as an off-label medication. 

### Azathioprine 

Azathioprine is an imidazole derivative of mercaptopurine. As a purine antagonist it acts as an antiproliferative agent, with its degradation products being incorporated as a false component into the DNA. The de novo synthesis of purines is essential for the proliferation of B and T cells, as these do not have a salvage pathway for DNA and RNA synthesis. The therapy with purine antagonists has a notable cytotoxic effect on B and T lymphocytes. In addition, a dose-dependent suppressive and cytotoxic effect of this agent to Langerhans cells could be shown in in vitro studies [37]. 

There are several studies on the use of this immunosuppressive agent in AD that could demonstrate an improvement of skin symptoms in children and adults. In a recent study the therapy with this agent lead to a significant improvement of skin symptoms in children after 3 months of administration, the tolerability was acceptable [26]. Furthermore, a significant reduction of total serum IgE was reached after 6 months [26]. 

Side effects of this therapy are gastrointestinal symptoms, hepatotoxicity and changes in the hemogram; even a severe bone marrow suppression can result. 

In some studies the occurrence of this fatal side effect could be avoided by previously determining the activity of the enzyme thiomethyl purine transferase (TMPT) and excluding those patients with a deficient activity of this enzyme which is important for the degradation of this agent [39]. 

The determination of TPMT activity could thus be used as a screening parameter for the selection of appropriate patients and if necessary for dose adjustment. 

In the UK and USA azathioprine is more frequently used in cases of severe AD than in Germany. Further studies should be carried out in order to learn more about the effect and side effect profile of this relatively low-cost agent. 

### Mycophenolate mofetil 

Mycophenolate mofetil (MMF), another purine antagonist, is a selective, non-competitive and reversible inhibitor of the enzyme inosine monophosphate dehydrogenase and inhibits the de novo synthesis of guanosine nucleotides [21]. 

MMF was originally isolated from a penicillium species and has, in addition to its antiproliferative effect, antibacterial and antimycotic properties [32]. In contrast to other immunosuppressive agents the toxicity of MMF is relatively low. 

Data concerning AD therapy with MMF are scarce. The case series that have so far been published show a good efficacy [4, 20, 21, 22, 40]; one female patient, however, experienced severe sepsis with subsequent endocarditis requiring mitral valve replacement [40, 43]. Thus, a careful risk-benefit assessment should be carried out before this agent is used off-label, and further studies concerning MMF therapy in severe AD should be carried out. 

### Methotrexate 

Methotrexate (MTX) is a folic acid antagonist which irreversibly inhibits dihydrofolate reductase and thus leads to a reduced production of tetrahydrofolate, an essential cofactor of purine and pyrimidine synthesis. Thus, MTX has an antiproliferative effect on T cells [2]. In addition, this immunosuppressive agent acts anti-inflammatory by increasing the intra- and extracellular adenosine concentration. Furthermore, MTX inhibits the chemotaxis of leukocytes as well as the release of multiple cytokines like TNF-α, IL-10 and IL-12, and there seems to be an additional, dose-independent effect on Langerhans cells [37]. 

Side effects are hepatotoxicity, nephrotoxicity, changes in the hemogram, increased susceptibility to infections and teratogenicity. In order to prevent severe side effects the hemogram, hepatitis serology and liver and kidney parameters should be analyzed and the folic acid concentration should be determined before the start of therapy. Furthermore, folic acid should be substituted once weekly during the course of therapy. 

For the treatment of Psoriasis vulgaris MTX has already been established as a safe and effective therapeutic [27]. Concerning the treatment of AD only scarce cases series exist. Present data are promising with regard to an improvement of skin symptoms in the treated patients [16, 19, 47, 54]. A recently published observational study could demonstrate that most patients with moderate-to-severe AD showed positive reactions after a 8- to 12-week MTX therapy and that tolerability was good [38]. The benefit was higher in adults than in children [38]. 

Placebo-controlled studies concerning MTX therapy in AD would be desirable in order to further evaluate this therapeutic approach. 

## Biologics 

Monoclonal antibodies, fusion proteins and recombinant human proteins, partly derived from rheumatology, have been successfully used for the treatment of severe therapy-resistant psoriasis for the last years. 

As psoriasis and AD are both T cell-mediated chronic inflammatory skin diseases and have several common characteristics, therapeutic intervention with biologics could also be considered for the treatment of severe refractory AD. Malignoma and a latent tuberculosis should be excluded before a therapy with biologics is started. 

In the chronic phase of AD, in which a Th1 immune response is prevailing, increased amounts of TNF-α could be detected in skin biopsies taken during therapy. 

The TNF-α antagonists etanercept and infliximab have so far only been used in few patients and could not provide convincing data [9, 29]. 

The monoclonal antibody mepolizumab inhibits IL-5, a cytokine that is important for the growth, differentiation and migration of eosinophils and thus plays an important role in AD. It could be shown that the use of this antibody in AD causes a reduction of the eosinophil count, but failed to achieve an improvement of the skin symptoms [41]. 

## Omalizumab 

Omalizumab is a monoclonal antibody that binds free IgE and thus prevents its binding to the high-affinity IgE receptor FcεRI so that it is not available for the induction of IgE-mediated allergic reactions. 

It could be demonstrated that this leads to a reduction of FcεRI expression on epidermal Langerhans cells [42]. The preparation is authorized for the therapy of severe bronchial asthma with a total IgE of up to 600 kU/l and is dosed depending on body weight and serum IgE. 

The results of clinical observational studies are inconsistent for AD. One study showed a response of AD to omalizumab [33], a further study demonstrated a response in approximately half of the patients [3]. Most of the studies, non of them placebo-controlled, showed positive results [18, 28, 34, 53]. 

One problem with the use of omalizumab in AD is the frequently very high serum IgE concentrations of the patients which do not allow for dosing according to the IgE-dependent manufacturers’ schemes. In the studies that have been carried out by now dosing was handled differently. Some authors applied the authorized maximum dose of 375 mg every 2 weeks [33, 34, 53], others increased the dose to 450 mg 2-times per month and others carried out a low-dose therapy with 150 mg every 2 weeks [3, 35]. 

In a recent observational study a significant improvement of the skin symptoms could be shown in 21 patients with AD and severe bronchial asthma, independently of their serum IgE [48]. 

### Rituximab 

Rituximab is a chimeric monoclonal antibody eliminating B cells by antibody-dependent cell-mediated cytotoxicity, complement-dependent toxicity or apoptosis. As CD20 is expressed by pre-B-cells and ripe B cells, but not by plasma cells, rituximab has no effect on the IgE-producing plasma cells. Therefore, the efficacy of this antibody cannot be explained by a reduction of specific antibodies [49]. 

A promising study on AD therapy with rituximab was carried out in which after 2 infusions of 1,000 mg rituximab each at a 2-week interval lead to a significant eczema improvement [49]. After therapy no B cells could be detected in the peripheral blood and the B cell count in the skin was reduced by 50%. Furthermore, after treatment reduced amounts of the Th2 cytokines IL-5 and IL-13 were detected in the skin, although the serum IgE concentrations did not change. 

### Allergen-specific immunotherapy 

Allergen-specific immunotherapy (SIT) is the only causal long-term therapy when sensitizations against allergens which represent important triggering factors in patients with extrinsic AD are present. In this context allergens from house dust mite are of particular importance. They are the most frequent perennial allergens and can cause a perennial aggravation of the skin condition in sensitized patients. 

In several studies the efficacy of specific immunotherapy in AD concerning the improvement of the skin condition could be demonstrated [12, 55]. Besides the clinical improvement, a reduction of serum factors correlated to the disease activity, like TARC/CCL17, CCL22 and IL-16, a decrease of allergen-specific IgE and an increase of serum IgG4 could be observed in treated patients [13]. Currently further prospective studies are carried out in order to find out whether AD alone, without concomitant allergic rhinitis or asthma, is an indication for SIT [15]. 

### Vitamin D3 

For AD it is known that the defective defense against microbial pathogens is caused, among other things, by a deficiency in antimicrobial peptides like cathelicidin, dermcidin or human β-defensins [23]. 

Considering that vitamin D3 is an important cofactor in the production of cathelicidin, AD patients were systemically treated with 40,000 IU cholecalciferol for 3 weeks. The authors could observe an up-regulation of cathelicidin in the skin of the patients; they did, however, not report if this treatment lead to a clinical improvement of AD [24]. 

## Conclusion and perspective 

For the therapy of severe AD only few alternatives exist when topic therapy does not lead to a stabilization of the clinical picture even after all possibilities, like consistent basic therapy, consistent use of local steroids and topic immunomodulators with a reactive or, latterly, proactive therapy regimen (i.e. application until the eczemas have healed, then application at the predilection sites 1- to 2-times weekly), photo therapy, patient education and so on, have been utilized. 

CyA, the only immunosuppressant that is market-authorized as a second-line therapy of AD, is not applicable in all patients due to its side effects. Further placebo-controlled studies would be necessary to establish alternative immunosuppressants, like MTX, azathioprine or MMF, in the therapy of AD. In the future also innovative therapies with monoclonal antibodies, like rituximab or omalizumab, could be a therapeutic option. Thymic stromal lymphopoietin (TSLP) is a further promising target, as a key role in the pathogenesis of AD is attributed to this IL-7-like cytokine. 

It is secernated by keratinocytes as a reaction to traumas and microbial antigens and allergens and leads to a strong stimulation of the pro-inflammatory Th2 immune response. Another therapeutic approach could be IL-31. A central role in pruritus development in AD is attributed to this cytokine, and in the mouse model an anti-IL-31-antibody lead to a reduction of scratching. Thus, we can be curious about future studies on new systemic AD therapies. 
